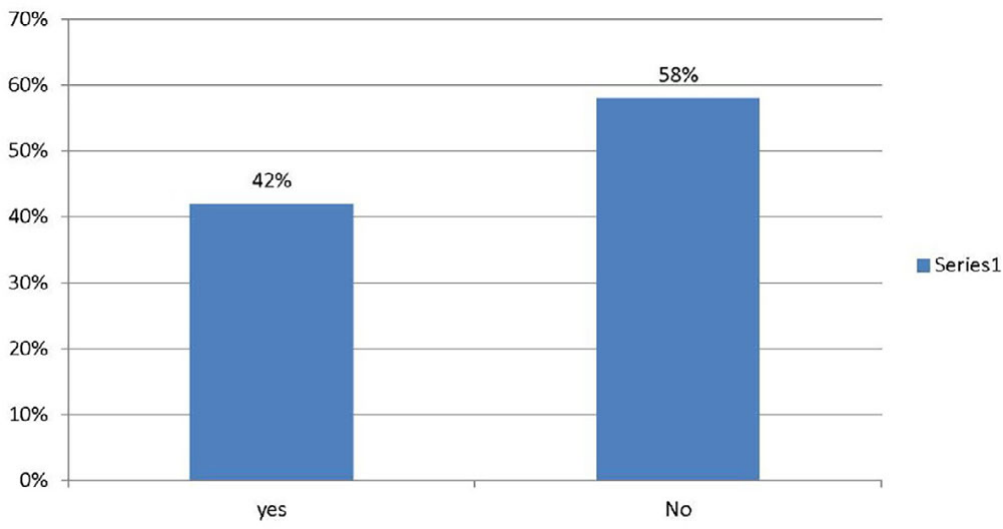# Capacity of the health facilities manage Alzheimer's and related dementia diseases in Uganda: Challenges and recommendations

**DOI:** 10.1002/alz70860_097496

**Published:** 2025-12-23

**Authors:** Abubakari Kiweewa

**Affiliations:** ^1^ African Research Center 4 Ageing & Dementia, Kampala, Central, Uganda

## Abstract

**Background:**

With the projected increase in number of older persons in both low and middle income countries, the burden of Alzheimer's and related dementia diseases (AD/ARDs) is projected to increase as well. However, the health systems inadequately prepared to offer optimal care for patients with AD/ARDs, despite the growing disease burden. Hence, the aim of this study was to assess the capacity of the health facilities to optimally manage Alzheimer's and related dementia diseases in Mukono district.

**Methods:**

We conducted a cross‐sectional between August and December 2018. A survey of 32 facilities (3 hospitals, 2 health center IV (HCIV), 15 health center III (HCIII) and 6 health center II (HCII) and 6 Private health facilities) in Mukono district. We conducted a thorough assessment of medical records, interviewed heads of the facilities and questionnaire was administered to 46 health workers. The study assessed the service provision for AD/ARDs, Knowledge of AD/ARDs management, challenges and opportunities.

**Results:**

Out of 32 health facilities assessed, 4 in 10 (42%) facilities reported managing (diagnosing/treating) clients with AD/ARDs, and majority (90.2%) were run by Non‐Physician Health Workers (NPHW). Only 2 in 10 of had guidelines for managing AD/ARDs. About less than half (46.4%) had AD/ARDs medicines in stocks (mainly Haloperidol) and all of the private facilities lacked essential medicine to treat AD/ARDs. All health center IIs lacked drugs for AD/ARDs. A significant knowledge gap in assessing and diagnosing AD/ARDs was observed among all the health workers. All health workers highlighted the need for addition training in AD/ARDs. Multitude of client and health provider challenges were observed in this study.

**Conclusion:**

Health facilities in Mukono district are inadequately prepared to offer optimal services for management of AD/ARDs. AD/ARDs drugs, knowledge gap and human resource for health presented a great challenge. In order to address the inadequately capacity to manage AD/ARDs, emphasis should be dwelt on strengthening the health facilities; including specialized training in assessment and diagnosis of AD/ARDS and improving the drugs and commodities supply chain in Uganda.